# Repeated Valproic Acid Administration Fundamentally Ameliorated Cisplatin-Induced Mechanical Allodynia in Rats

**DOI:** 10.3390/ijms26114977

**Published:** 2025-05-22

**Authors:** Yoshihiro Seto, Yuki Ohara, Manami Tachi, Mari Tomonari, Daisuke Inoue, Fumiyasu Okazaki, Yasuhiro Tsuji, Hideto To

**Affiliations:** 1 Department of Medical Pharmaceutics, Graduate School of Medicine and Pharmaceutical Sciences, University of Toyama, Toyama 930-0194, Japandinoue@pha.u-toyama.ac.jp (D.I.); f-okazaki@daiichi-cps.ac.jp (F.O.); hidetoto@pha.u-toyama.ac.jp (H.T.); 2 Department of Drug Analysis, Daiichi University of Pharmacy, Fukuoka 815-8511, Japan; 3 Laboratory of Clinical Pharmacometrics, School of Pharmacy, Nihon University, Chiba 274-8555, Japan; tsuji.yasuhiro@nihon-u.ac.jp

**Keywords:** valproic acid, cisplatin, mechanical allodynia

## Abstract

Cisplatin (cis-diamminedichloro-platinum; CDDP) is a chemotherapeutic agent that frequently induces peripheral neuropathy characterized by mechanical allodynia. Herein, we aimed to determine the effects of valproic acid (VPA) on cisplatin-induced mechanical allodynia in rats and elucidate the underlying mechanisms. A single administration of VPA (150 mg/kg) transiently suppressed CDDP-induced mechanical allodynia, correlating with serum VPA concentrations. Repeated VPA administration before or after the onset of CDDP-induced mechanical allodynia significantly attenuated allodynia even after VPA discontinuation, suggesting fundamental treatment potential. Mechanistically, CDDP increased the expression of neurokinin 1 receptor (NK1R) mRNA in the dorsal horn of the spinal cord, and this increased expression was suppressed by repeated VPA administration. Treatment with an NK1R antagonist alleviated CDDP-induced mechanical allodynia, indicating the involvement of NK1R in allodynia. In vitro assays revealed that VPA did not affect the cytotoxicity of CDDP in Walker 256 cells, suggesting that VPA does not interfere with the antitumor activity of CDDP. Overall, repeated VPA administration may fundamentally ameliorate CDDP-induced peripheral neuropathy by suppressing the CDDP-induced increased NK1R expression without compromising the antitumor effects of CDDP. These findings provide insights into the potential use of VPA as a therapeutic agent for managing CDDP-induced peripheral neuropathy.

## 1. Introduction

Cisplatin, cis-diamminedichloro-platinum (CDDP), is widely used to treat non-small cell lung, head and neck, ovarian, and breast cancer [1,2,3,4]. Although CDDP exhibits robust therapeutic effects and is the primary drug used for cancer chemotherapy, treatment with CDDP is frequently associated with the development of peripheral neuropathy, a dose-limiting adverse effect of CDDP [5,6]. Although improvement in CDDP-induced peripheral neuropathy has been reported in both basic and clinical settings [7,8,9,10,11], treatment for CDDP-induced peripheral neuropathy is yet to be established.

CDDP-induced peripheral neuropathy is characterized by mechanical allodynia as the initial symptom, with sensory insensitivity reported in severe cases [12,13]. Patients with CDDP-induced mechanical allodynia experience pain due to non-nociceptive stimuli, such as the rustling of clothes, have a reduced quality of life, and encounter difficulties in undergoing continued cancer chemotherapy with CDDP [12,13,14]. Moreover, CDDP-induced mechanical allodynia is frequently resistant to available analgesics [15,16], necessitating the development of effective drugs to treat CDDP-induced peripheral neuropathy.

Valproic acid (VPA) is widely used in the treatment of epilepsy [17], as well as manic-depressive psychosis and migraine headaches [18,19]. Basic studies have revealed that VPA can ameliorate the peripheral neuropathy caused by nerve injury and diabetes [20,21]. For treating epilepsy, manic-depressive psychosis, and migraine headaches, the mechanism of VPA involves increased gamma-aminobutyric acid (GABA) levels, sodium channel inhibition, and calcium channel inhibition [22]. Recently, VPA was found to possess histone deacetylase (HDAC) inhibitory properties, and these HDAC inhibitory effects of VPA reportedly protect the retinal and sciatic nerves [23,24,25]. Thus, we speculate that VPA can ameliorate CDDP-induced mechanical allodynia and could be a fundamental treatment for CDDP-induced mechanical allodynia.

In this study, we examined the effects of VPA on CDDP-induced mechanical allodynia and elucidated the mechanisms of VPA in CDDP-induced mechanical allodynia in rats.

## 2. Results

### 2.1. Dose–Response Effects and Temporal Changes on CDDP-Induced Mechanical Allodynia in Rats Following Single Administration of VPA

**Figure 1:**
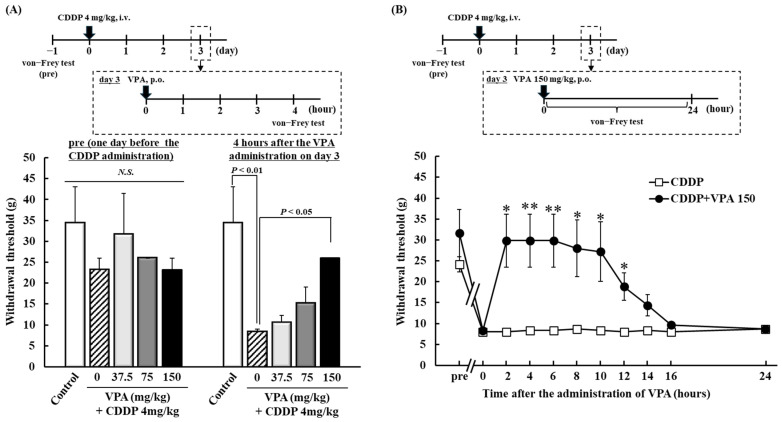
Dose–response effects and temporal changes after a single administration of VPA on CDDP-induced mechanical allodynia in rats. (**A**) CDDP (4 mg/kg) was intravenously administered at 5:00 on day 0, with saline administered to the control group. VPA (75, 150, or 300 mg/kg/day) was orally administered at 8:00 on day 3, and the von Frey test was performed on days −1 and 3. Each value represents the mean and standard error (SEM; *n* = 4). (**B**) CDDP (4 mg/kg) was intravenously administered at 5:00 on day 0, and VPA (150 mg/kg) was orally administered at 8:00 on day 3. The von Frey test was performed 2, 4, 6, 8, 10, 12, 14, 16, and 24 h after the VPA administration. Each value represents the mean and SEM (*n* = 6). *: *p* < 0.05, **: *p* < 0.01, N.S. means not significant among all groups. CDDP, cisplatin (cis-diamminedichloro-platinum); VPA, valproic acid.

Following drug pretreatment (day −1; Figure 1A), withdrawal thresholds did not significantly differ between groups. Mechanical allodynia was examined in rats three days after CDDP administration, and the effects of a single dose of VPA (37.5, 75, or 150 mg/kg) on CDDP-induced mechanical allodynia were assessed (Figure 1A). Four hours after VPA administration, the CDDP group (CDDP + VPA 0 mg/kg) had significantly lower withdrawal thresholds than the control group (*p* < 0.01). In the VPA group, the withdrawal threshold of the CDDP + VPA 150 mg/kg group was significantly higher than that of the CDDP group (*p* < 0.05). In addition, we determined temporal changes in the effects of VPA 150 mg/kg on CDDP-induced mechanical allodynia. At 2, 4, 6, 8, 10, and 12 h after administering 150 mg/kg of VPA, the CDDP + VPA150 group exhibited significantly higher withdrawal thresholds than the CDDP group (*p* < 0.05 and 0.01, respectively; Figure 1B).

### 2.2. Effect of Repeated VPA Administration on CDDP-Induced Mechanical Allodynia in Rats

**Figure 2:**
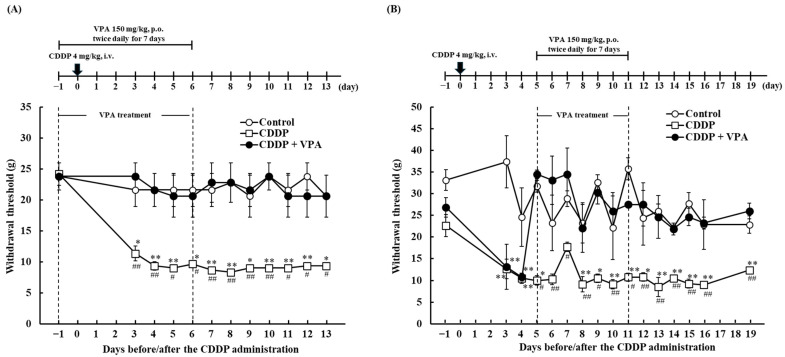
Effect of repeated administration of VPA on CDDP-induced mechanical allodynia in rats. (**A**) CDDP (4 mg/kg) or saline was intravenously administered at 5:00 on day 0. VPA (150 mg/kg) or simple syrup was orally administered twice daily (8:00 and 20:00) for 7 days (day −1 to day 6). Each value represents the mean and standard error (SEM; *n* = 5–6). *: *p* < 0.05, **: *p* < 0.01 vs. control; #: *p* < 0.05, ##: *p* < 0.01 vs. CDDP + VPA. (**B**) CDDP (4 mg/kg) or saline was intravenously administered at 5:00 on day 0. VPA (150 mg/kg) or simple syrup was orally administered twice daily (8:00 and 20:00) for 7 days (days 5 to 11). Each value represents the mean and SEM (*n* = 7–8). *: *p* < 0.05, **: *p* < 0.01 vs. control; #: *p* < 0.05, ##: *p* < 0.01 vs. CDDP + VPA. CDDP, cisplatin (cis-diamminedichloro-platinum); VPA, valproic acid.

To evaluate the effect of repeated VPA administration on CDDP-induced mechanical allodynia, VPA was repeatedly administered to the experimental rats twice daily before CDDP administration or after the onset of CDDP-induced mechanical allodynia. Upon administering VPA from days −1 to 6, no significant differences in withdrawal thresholds were observed between the control and CDDP + VPA groups on days 3–6 during VPA administration (Figure 2A). The withdrawal threshold of the CDDP + VPA group did not significantly differ from that of the control group on days 7–13 after discontinuation of VPA administration. The CDDP group had significantly lower withdrawal thresholds than the control and CDDP + VPA groups on days 3–13, respectively (*p* < 0.05 and 0.01, respectively; Figure 2A).

Next, we examined the effects of repeated VPA administration on the development of CDDP-induced mechanical allodynia. The CDDP group had a significantly lower withdrawal threshold than the control group on days 3, 4, 5, 8, 9, 10, 11, 12, 13, 14, 15, 16, and 19 (*p* < 0.05 and 0.01, respectively; Figure 2B). On days 3 and 4, prior to VPA treatment, the CDDP + VPA group exhibited a significantly lower withdrawal threshold than the control group (*p* < 0.01). Upon VPA administration from days 5 to 11, the withdrawal thresholds did not differ significantly between the CDDP + VPA group and the control and CDDP + VPA groups on days 5–11 during VPA administration. Furthermore, as shown in Figure 2A, there was no significant difference in the withdrawal thresholds between the control and CDDP + VPA groups from day 12 to 19 following the termination of VPA administration. Meanwhile, the withdrawal thresholds of the CDDP + VPA group were significantly lower than those of the CDDP group from day 5, the start of VPA administration, to day 19 (*p* < 0.05 and 0.01, respectively).

### 2.3. Time Profiles of Serum VPA Concentrations Following Single and Repeated VPA Administration in Rats

In patients with epilepsy, VPA is typically subjected to therapeutic drug monitoring (TDM) and is positively correlated with serum concentration and effectiveness [26,27]. Next, we assessed the serum concentrations of VPA after single and repeated VPA administrations. Fourteen hours after VPA administration, the VPA concentration was below the detection limit of the assay used in this study (Table 1). For repeated administration, VPA was administered twice daily for 14 times. The serum concentration measured after the final administration was below the detection limit and was undetectable 72 h after VPA administration (Table 2).

### 2.4. Effects of Single and Repeated VPA Administration on Nk1r mRNA in Spinal Cord Dorsal Horn of Rats

**Figure 3:**
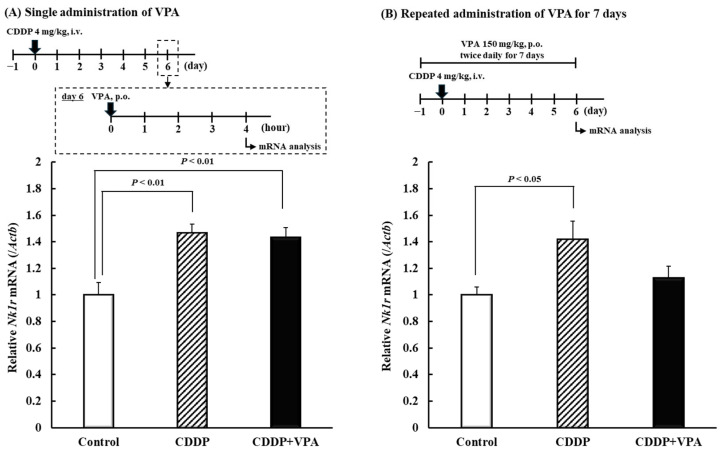
Effects of single and repeated VPA administration on Nk1r mRNA expression in the spinal cord dorsal horn of rats. (**A**) Expression of *Nk1r* mRNA in the spinal cord dorsal horn after a single administration of VPA. CDDP (4 mg/kg) or saline was intravenously administered at 5:00 on day 0. VPA (150 mg/kg) or simple syrup was orally administered at 8:00 on day 6. Each value represents the mean and standard error (SEM; *n* = 6). (**B**) Expression of *Nk1r* mRNA level in the spinal cord dorsal horn after repeated VPA administration. CDDP (4 mg/kg) or saline was intravenously administered at 5:00 on day 0. VPA (150 mg/kg) or simple syrup was orally administered twice daily (8:00 and 20:00) for 7 days (day −1 to day 6). Each value represents the mean and SEM (*n* = 6). CDDP. CDDP, cisplatin (cis-diamminedichloro-platinum); VPA, valproic acid.

Neurokinin 1 receptor (NK1R) reportedly plays a role in chronic pain, including mechanical allodynia [28,29]. Accordingly, we detected expression levels of *Nk1r* mRNA on day 6 after single or repeated administration of VPA. Four hours after a single VPA administration, the CDD-only and CDDP + VPA groups exhibited significantly higher *Nk1r* mRNA expression levels than the control group (*p* < 0.01; Figure 3A). However, 4 h after the final administration of repeated doses, the CDDP-only group exhibited significantly increased *Nk1r* mRNA expression compared with the control group (*p* < 0.05; Figure 3B). Upon repeated VPA administration, expression of *Nk1r* mRNA did not differ significantly between the control and CDDP + VPA groups.

### 2.5. Effect of NK1R Antagonist on CDDP-Induced Mechanical Allodynia in Rats

**Figure 4:**
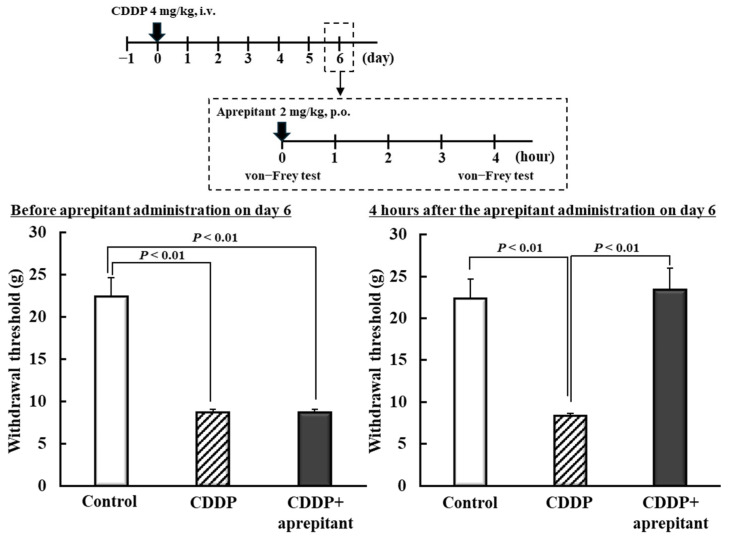
Effects of NK1R antagonist on CDDP-induced mechanical allodynia in rats. CDDP (4 mg/kg) or saline was intravenously administered at 5:00 on day 0. An NK1R antagonist, aprepitant (2 mg/kg), or vehicle was orally administered at 8:00 on day 6. The von Frey test was performed 4 h after aprepitant administration. Each value represents the mean and standard error (SEM; *n* = 6). CDDP. CDDP, cisplatin (cis-diamminedichloro-platinum); VPA, valproic acid.

Next, we determined whether CDDP-induced mechanical allodynia can be improved by inhibiting NK1R expression, which increased in the spinal cord dorsal horn during CDDP administration (Figure 3). Six days after CDDP administration, the group treated with aprepitant (2 mg/kg), an NK1R antagonist, showed a significant increase in the withdrawal threshold compared with the CDDP group (*p* < 0.01; Figure 4).

### 2.6. Effect of VPA Administration on Tumor Cell Viability

**Figure 5:**
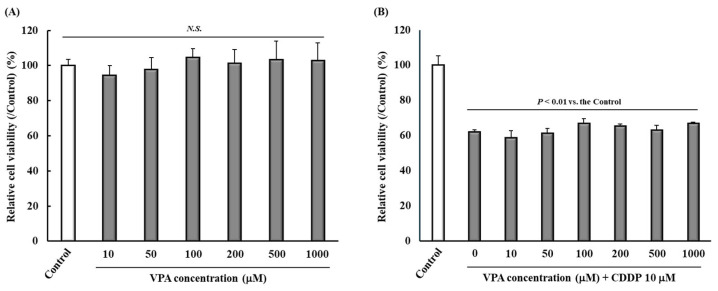
Effect of VPA on the viability of tumor cells. Walker 256 cells were incubated with VPA (**A**) or CDDP 10 μM + VPA (**B**) for 24 h, and cell viability was assessed. Each value represents the mean and standard error (SEM; **A**: *n* = 6, **B**: *n* = 3). N.S. means not significant among all groups. CDDP, cisplatin (cis-diamminedichloro-platinum); VPA, valproic acid.

At concentrations of 10, 50, 100, 200, 500, and 1000 μM, VPA did not affect the viability of Walker 256 cells, a rat cell line derived from mammary carcinoma (Figure 5A). CDDP (10 μM) significantly reduced the viability of Walker 256 cells compared with the Control. However, there were no significant differences among groups administered CDDP (10 μM) plus each VPA concentration (Figure 5B).

## 3. Discussion

CDDP-induced peripheral neuropathy often persists even after treatment discontinuation [1,2,3]. Currently, the mechanism underlying CDDP-induced peripheral neuropathy remains unclear, and there is no established treatment for this condition in clinical settings [15,16,30]. In the current study, we determined whether VPA could fundamentally improve CDDP-induced peripheral neuropathy in experimental rats.

Initially, we examined the effects of a single dose of VPA on CDDP-induced mechanical allodynia. VPA 150 mg/kg transiently suppressed CDDP-induced mechanical allodynia from 2 to 12 h after administration of VPA alone (Figure 1B). Plasma concentrations of VPA increased rapidly from 0.25 h post-dose and were detectable until 12 h post-dose when allodynia was suppressed after a single dose (Table 1). Furthermore, VPA was not detected in the serum 14 h after a single administration when no anti-allodynic effects were observed (Figure 1B) (Table 1). VPA has been shown to suppress neuropathic pain in a mouse model of spinal nerve ligation, correlating with blood concentration trends [31]. In addition, TDM is typically performed for patients with epilepsy undergoing treatment with VPA, and there exists a correlation between serum TDM concentration and the efficacy of VPA [26,27]. Thus, the anti-allodynic effect of a single administration of VPA could depend on the serum concentration of VPA, and a rapid anti-allodynic effect after a single dose may be very useful for patients with CDDP-induced allodynia.

Next, we investigated the effects of repeated VPA administration on CDDP-induced mechanical allodynia before and after the onset of allodynia. Regarding the dosing schedule, VPA was administered twice every 12 h daily (8:00 and 20:00) because 150 mg/kg VPA effectively suppressed CDDP-induced mechanical allodynia from 2 to 12 h after a single administration (Figure 1B). Herein, we found that administration of VPA (150 mg/kg) twice daily for 7 days prior to CDDP administration suppressed CDDP-induced mechanical allodynia, both during VPA administration and after VPA discontinuation (Figure 2A). Moreover, administration of VPA for 7 days after the onset of CDDP-induced mechanical allodynia could suppress CDDP-induced mechanical allodynia from the day of VPA initiation, as well as after VPA discontinuation (Figure 2B). Following repeated administration, serum concentrations were undetectable 72 h (3 days) after VPA administration (Table 2). Therefore, repeated VPA administration suppressed CDDP-induced mechanical allodynia even when the serum VPA concentration was undetectable, and the mechanism underlying the anti-allodynic effect differed between single and repeated administration of VPA. In an in vitro study, VPA reportedly improved the cell viability of a CDDP-treated primary culture of rat dorsal root ganglion [32]. Additionally, inhibition of HDAC, an identified VPA-mediated action, regulates pain-related factors [33]. In the present study, HDAC activity was not assessed. However, HDAC inhibition has previously been reported in rats at doses of 200–400 mg/kg/day of VPA [34,35]. Therefore, we consider that the present VPA dosage of 300 mg/kg/day shows HDAC inhibition. We previously reported that the CDDP dosing schedule used in this study did not cause histological damage [36], and hence, we focused on the role of NK1R in the chronicity of pain in the current study.

Peripheral/central sensitization contributes to the development of CDDP-induced mechanical allodynia [37,38]. Peripheral sensitization is a state of increased sensitivity in primary neurons and is thought to be caused by increased expression and function of voltage-gated sodium and calcium channels [39,40]. VPA is known to suppress sodium and calcium channels, and a single dose could exert an immediate anti-allodynic effect by directly inhibiting these channels [22]. During repeated administration of VPA, CDDP-induced mechanical allodynia presumably improved owing to the inhibition of these channels in the presence of VPA. Following repeated VPA administration, the anti-allodynic effect persisted even after VPA was undetectable in the serum. Therefore, suppression of CDDP-induced mechanical allodynia by repeated VPA administration suggests an association other than peripheral sensitization. Central sensitization is a state of increased sensitivity in secondary neurons [39]. NK1R plays a major role in central sensitization [39,40]. VPA reportedly suppressed NK1R expression in U373 MG human astrocytoma cells in vitro [41]. Therefore, we detected the expression of *Nk1r* mRNA in the spinal cord dorsal horn of rats. Expression of *Nk1r* mRNA was elevated in rats exhibiting CDDP-induced mechanical allodynia, and repeated administration of VPA, rather than a single dose, suppressed Nk1r mRNA (Figure 3A,B). Furthermore, we found that treatment with an NK1R antagonist suppressed CDDP-induced mechanical allodynia (Figure 4). CDDP was shown to increase *Nk1r* mRNA levels in the tracheal bronchus [42]. Moreover, NK1R was found to be involved in chronic pain in mouse models [43]. VPA, when administered as a single dose, transiently suppressed CDDP-induced mechanical allodynia despite not suppressing NK1R elevation. The mechanism by which VPA suppressed CDDP-induced mechanical allodynia during a single dose may be through increased GABA and sodium/calcium channel inhibition. Therefore, various factors such as GABA, sodium/calcium channels, and NK1 are involved in CDDP-induced mechanical allodynia, suggesting that it is important to suppress factors other than NK1R in terms of pain suppression. Additionally, NK1R potentially contributed to CDDP-induced mechanical allodynia in this study, and repeated VPA administration could offer a fundamental treatment for CDDP-induced mechanical allodynia owing to the suppression of NK1R expression. Therefore, we propose, in the present study, that repeated dosing of VPA over a period of time is important, even when the pathogenesis of CDDP-induced mechanical allodynia is taken into account. Among the several VPA-mediated effects, we speculate that HDAC inhibition affects NK1R expression, and we plan to clarify this possibility in future investigations.

When a drug is co-administered with an anticancer drug such as CDDP, it is crucial that it does not interfere with antitumor activity, that is, the main action of the anticancer drug [44]. Finally, we demonstrated that VPA did not affect the cytotoxic effects of CDDP in Walker 256 cells in vitro (Figure 5B). VPA-mediated HDAC inhibition reportedly exerts antitumor effects [45]. Therefore, VPA did not suppress the antitumor effects of CDDP. Inhibition of HDACs has been reported to affect cellular functions such as muscle exhaustion and short chain fatty acids, which are involved in various metabolic processes, and sirtuin-3, which is involved in neuroprotection and aging [46,47,48]. The present study focused on the antitumor effects of CDDP and evaluated its effect on cell viability. Future work on the effects of VPA on cellular functions of normal cells at the cellular and biological levels will further support the usefulness of VPA in CDDP-induced allodynia.

In conclusion, repeated administration of VPA could ameliorate CDDP-induced peripheral neuropathy when administered before or after the onset of neuropathy. Regarding the mechanism of action, VPA suppressed CDDP-induced mechanical neuropathy by inhibiting the CDDP-induced increase in NK1R expression.

## 4. Materials and Methods

### 4.1. Animals

Male Sprague Dawley rats were purchased from Japan SLC, Ltd. (Hamamatsu, Japan). Two to three rats were housed per cage under standard light–dark cycle conditions (lights on and off at 7:00 and 19:00, respectively) at 23–25 °C and 50–60% relative humidity, with free access to food and water. All mice were acclimatized for one week before experimentation.

All animal procedures were approved by the Ethics Committee of Animal Experiments in accordance with the Internal Regulations of the University of Toyama Animal Experiment Committee (approval numbers: A2014PHA-3, A2017PHA-15, and A2020PHA-25), which are based on the Law for Humane Treatment and Management of Animals (Law No. 105 on 1 October 1973, amended on 2 June 2017).

### 4.2. Cell Culture and Drugs

Walker 256 cells, a rat mammary gland carcinoma cell line, were obtained from the Cell Resource Center for Biomedical Research at Tohoku University (Sendai, Japan). The cells were cultured in RPMI 1640 medium (Nacalai Tesque, Inc., Kyoto, Japan) supplemented with 10% Gibco fetal bovine serum (Thermo Fisher Scientific, Waltham, MA, USA) at 37 °C in a humidified atmosphere containing 5% CO_2_. Subcultures were performed every 2–3 days until the cells reached confluence, and the total number of subcultures did not exceed 15.

CDDP was procured by Nippon Kayaku Co., Ltd. (Tokyo, Japan) and dissolved in saline. Depakene syrup (5%) was purchased from Kyowa Kirin Co., Ltd. (Tokyo, Japan) and diluted in a simple syrup for the animal experiments. Aprepitant was purchased from Tokyo Chemical Industry Co., Ltd. (Tokyo, Japan) and suspended in dimethyl sulfoxide and corn oil to 0.4 mg/mL (final concentration of 2.5%) for animal experiments. Sodium valproate and diazepam were purchased from FUJIFILM Wako Pure Chemical Corporation (Osaka, Japan) for the MTT (3-[4,5-dimethylthiazol-2-yl]-2,5 diphenyl tetrazolium bromide) assay and high-performance liquid chromatography (HPLC) analysis.

### 4.3. Experiment I: Dose–Response Effects and Temporal Changes on CDDP-Induced Mechanical Allodynia in Rats Following Single VPA Administration

CDDP (4 mg/kg) was administered intravenously at 5:00 on day 0; the control group was administered the vehicle. Three days after CDDP administration (day 3), VPA (37.5, 75, or 150 mg/kg) was orally administered at 8:00, with the vehicle administered to the control group. To assess mechanical allodynia, the von Frey test was performed 6 h after VPA administration. To evaluate the time profile of the effect of VPA on CDDP-induced mechanical allodynia, VPA (150 mg/kg) was administered orally at 8:00, and the von Frey test was performed 2, 4, 6, 8, 10, 12, 14, 16, and 24 h after VPA administration.

### 4.4. Experiment II: Effects of Repeated VPA Administration on CDDP-Induced Mechanical Allodynia in Rats

CDDP (4 mg/kg) was administered intravenously at 5:00 on day 0, with the vehicle administered to the Control group. For prophylactic treatment, VPA (150 mg/kg) was administered orally twice daily from day −1 to day 6. Control group rats were administered the vehicle. Following the induction of CDDP-induced mechanical allodynia, VPA (150 mg/kg) was orally administered twice daily from days 5 to 11. The vehicle was administered to rats in the control group. The von Frey test was performed to assess mechanical allodynia before and after CDDP administration.

### 4.5. Experiment III: Time Profiles of Serum Concentration After Single and Repeated VPA Administration in Rats

For assessing single administration, VPA (150 mg/kg) was administered orally at 8:00, and blood samples (about 100–200 μL) were collected from the tail vein following 5 min of restraint in a holding device at 0.25 h, 0.5 h, 1 h, 2 h, 4 h, 6 h, 8 h, 12 h, 14 h, 16 h, and 24 h after VPA administration. For assessing repeated administration, VPA (150 mg/kg) was orally administered twice daily 14 times, and blood samples (about 100–200 μL) were collected from the tail vein following 5 min of restraint in a holding device at 0.25 h, 0.5 h, 1 h, 2 h, 4 h, 6 h, 8 h, 12 h, 24 h, 48 h, and 72 h after the final VPA administration (at 8:00). With reference to previous reports [49], the total blood volume collected was not to exceed 15% of the circulating blood volume of rat. All blood samples were centrifuged at 3500× *g* for 5 min at 15 °C, and serum was collected.

To detect serum VPA concentrations, HPLC analysis was performed as described previously [50]. Each serum sample (50–100 μL) was added to 250–500 μL of acetonitrile containing diazepam (internal standard) and vortexed for 1 min. After standing for 10 min, the mixed liquid was centrifuged at 12,000× *g* for 10 min at 15 °C, and the supernatant was concentrated and centrifuged (room temperature, 5.1 Torr, 2.5 h) using a Thermo Scientific Savant SpeedVac SPD1010 (Thermo Fisher Scientific). Thereafter, 150 μL of the mobile phase was added, and VPA concentrations were detected using an HPLC system with ultraviolet (UV) detection (Shimadzu Corporation, Kyoto, Japan). The assay conditions were as follows: column: Unison US-C18 (Imtakt Corporation, Kyoto, Japan); mobile phase, 50 mM phosphate buffer (pH 3.0)–acetonitrile (55:45); flow rate, 1.2 mL/min; injection volume, 50 μL; column oven, 50 °C; and UV wavelength, 210 nm.

### 4.6. Experiment IV: Effects of Single and Repeated VPA Administration on Nk1r mRNA Expression in Spinal Cord Dorsal Horn and Effect of NK1R Antagonist on CDDP-Induced Mechanical Allodynia in Rats

For assessing single administration, CDDP (4 mg/kg) was administered intravenously at 5:00 on day 0, and the vehicle was administered to the control group. VPA (150 mg/kg) was administered orally at 8:00, and the spinal cord dorsal horn was collected 4 h after VPA administration. For assessing repeated administration, VPA (150 mg/kg) was orally administered twice daily for 14 days, and the spinal cord dorsal horn was collected 4 h after the final VPA administration (at 8:00). Total RNA was extracted using RNAiso Plus (TaKaRa Bio Inc., Kusatsu, Japan), and cDNA was synthesized using the PrimeScript RT Reagent Kit with gDNA Eraser (TaKaRa Bio Inc.). Real-time PCR was performed using the KOD SYBR qPCR Mix (TOYOBO, Kita, Japan) with StepOnePlus (Thermo Fisher Scientific) to analyze *Nk1r* mRNA expression. *Actb* was employed as the housekeeping gene, and the mean value of the lowest expression level was standardized to 1. The primer sequences are shown in Table S1.

Aprepitant was used as the NK1R antagonist. CDDP (4 mg/kg) was administered intravenously at 5:00 on day 0, with the control group administered the vehicle. Aprepitant (2 mg/kg) was orally administered at 8:00 h on day 6, and the von Frey test was performed to assess mechanical allodynia 4 h after aprepitant administration.

### 4.7. Experiment V: Effect of VPA on Tumor Cell Viability

In brief, Walker 256 cells were seeded at a density of 2 × 10^3^ cells/100 μL in each well of a 96-well plate and preincubated for 24 h. After pre-incubation, CDDP (final concentration: 10 μM) and VPA (final concentrations: 10, 20, 30, 40, and 50 μM) were added to the cell culture medium and incubated for 24 h. Following incubation with CDDP and VPA, the MTT Cell Count Kit (Nacalai Tesque, Inc.) was used to assess cell viability.

### 4.8. Von Frey Test

The von Frey test was performed as described previously [51,52]. Mechanical allodynia was assessed using a touch-test sensory evaluator (Muromachi Kikai Co., Ltd., Tokyo, Japan). Individual rats were placed in a plastic cage with a wire mesh floor and allowed to acclimate for 10 min before the hind paw mechanical thresholds were measured. Filaments with bending forces ranging between 1 and 60 g were applied to the middle of the plantar surface of the right hind paw and held for 5 s. The withdrawal threshold of the right hind paw was assessed by increasing the stimulus strength from the 1 g filament until paw withdrawal occurred.

### 4.9. Statistical Analysis

All values are presented as mean ± standard error of the mean (SEM). One-way analysis of variance was used for multiple comparisons, followed by Scheffé’s or Dunnett’s test to compare two groups. Student’s *t*-test was used for comparison between pairs of groups. Statistical significance was set at *p* < 0.05. All statistical analyses were performed using SPSS software version 29 (RRID:SCR_ 002865).

While conducting the von-Frey test during the in vivo study, the number of samples varied because individuals who were not stable in the von Frey test were excluded. Although no statistical method was used to predetermine sample sizes, the effect size was confirmed in each experiment, ensuring statistical validity (Supplementary Data S2).

## Figures and Tables

**Table 1:** Time profile of the serum VPA concentration (mg/mL) after administration in rats.

Time After a Single VPA Administration										
0.25 h	0.5 h	1 h	2 h	4 h	6 h	8 h	12 h	14 h	16 h	24 h
77.09 ± 6.01	65.62 ± 5.77	38.20 ± 5.94	10.28 ± 1.47	18.81 ± 2.05	20.07 ± 1.99	13.51 ± 0.95	8.62 ± 1.49	N.D.	N.D.	N.D.

VPA (150 mg/kg) was administered orally at 8:00. VPA is not detected 14 h after administration. Each value represents the mean and standard error (SEM; *n* = 5). N.D., not detected; VPA, valproic acid.

**Table 2:** Time profile of the serum VPA concentration (mg/mL) after repeated administration in rats.

Time After Repeated VPA Administration										
0.25 h	0.5 h	1 h	2 h	4 h	6 h	8 h	12 h	24 h	48 h	72 h
75.98 ± 12.36	56.99 ± 7.06	20.32 ± 3.39	9.36 ± 2.21	17.37 ± 2.67	19.00 ± 2.65	15.27 ± 2.27	16.59 ± 0.68	6.56 ± 2.30	1.78 ± 1.75	N.D.

VPA (150 mg/kg) was orally administered twice daily (8:00 and 20:00), 14 times (final VPA administration at 8:00). Each value represents the mean and standard error (SEM; *n* = 5). N.D., not detected; VPA, valproic acid.

## Data Availability

The data presented in this study are available on request from the corresponding author.

## Supplementary Materials

The following supporting information can be downloaded at: https://www.mdpi.com/article/10.3390/ijms26114977/s1.

## Abbreviations

CDDP	Cis-diamminedichloro-platinum
GABA	Gamma-aminobutyric acid
HPLC	High-performance liquid chromatography
NK1R	Neurokinin 1 receptor
SEM	Standard error of the mean
TDM	Therapeutic drug monitoring
UV	Ultraviolet
VPA	Valproic acid
