# Supplementary material for: Repeated Valproic Acid Administration Fundamentally Ameliorated Cisplatin-Induced Mechanical Allodynia in Rats
Source: Int J Mol Sci. 2025 May 22;26(11):4977. doi: 10.3390/ijms26114977 (PMC12155109; doi:10.3390/ijms26114977)
Supplement: Supplementary file 1 [file ijms-26-04977-s001.zip › VPA supplementary data revise1/VPA_reduce_CDDP_PN_supplementary_data1.pdf]

Table S1 PCR Primers

Gene name	Forward	Reverse
Rat *Nk1r*	AACCCCATCATCTACTGTTGCC	TTTCCAGCCCCTCATAATCACC
Rat *Actb*	GTCATCACTATCGGCAATGAGC	GTAGTTTCATGGATGCCACAGG

NCBI accession numbers for each target: Rat *Nk1r* (NM_012667.2) *Actb* (NM_031144.3)
