# Supplementary material for: Repeated Valproic Acid Administration Fundamentally Ameliorated Cisplatin-Induced Mechanical Allodynia in Rats
Source: Int J Mol Sci. 2025 May 22;26(11):4977. doi: 10.3390/ijms26114977 (PMC12155109; doi:10.3390/ijms26114977)
Supplement: Supplementary file 1 [file ijms-26-04977-s001.zip › VPA supplementary data revise1/VPA_reduce_CDDP_PN_supplementary_data2.pdf]

Supplementary data 2: the effect size of each experiment

Figure 1A

	pre	day 3
η^2	**0.161**	**0.643**

Figure 1B

	pre	0	2 h	4 h	6 h	8 h	10 h	12 h	14 h	16 h	24 h
**Cohen d**	**0.727**	**0.577**	**2.002**	**1.969**	**1.969**	**1.646**	**1.520**	**1.878**	**1.353**	**1.199**	**0**

Figure 2A

	day -1	day 3	day 4	day 5	day 6	day 7	day 8	day 9	day 10	day 11	day 12	day 13
η^2	**0.002**	**0.635**	**0.650**	**0.591**	**0.565**	**0.660**	**0.655**	**0.591**	**0.805**	**0.591**	**0.660**	**0.504**

Figure 2B

	day -1	day 3	day 4	day 5	day 6	day 7	day 8	day 9	day 10	day 11	day 12	day 13	day 14	day 15	day 16	day 19
η^2	**0.054**	**0.513**	**0.810**	**0.418**	**0.481**	**0.273**	**0.603**	**0.407**	**0.793**	**0.444**	**0.418**	**0.929**	**0.552**	**0.503**	**0.740**	**0.753**

Figure 3

	(A) Single administration of VPA	(B) Repeated administration of VPA
η^2	**0.598**	**0.375**

Figure 4

	Before aprepitant administration on day 6	4 hours after the aprepitant administration on day 6
η^2	**0.813**	**0.691**

Figure 5

	Figure 5A	Figure 5B
η^2	**0.039**	**0.889**
